# Plasminogen Activator Inhibitor-1 is Internalized by Endothelial Cells via A Pinocytosis-Like Mechanism and Evades Degradation

**DOI:** 10.1096/fj.202600061R

**Published:** 2026-05-31

**Authors:** Molly McAdow, Gjina Ahmetaj, Rachel Hauschel, Anne Eichmann, William Sessa

**Affiliations:** 1 Department of Obstetrics, Gynecology, & Reproductive Sciences, Yale School of Medicine, New Haven, Connecticut, USA; 2 Department of Molecular and Cellular Physiology, Yale University School of Medicine, New Haven, Connecticut, USA; 3 Cardiovascular Research Center, Department of Internal Medicine, Yale University School of Medicine, New Haven, Connecticut, USA; 4 Paris Cardiovascular Research Center, Inserm U970, Université Paris, Paris, France; 5 Department of Pharmacology, Yale University School of Medicine, New Haven, Connecticut, USA

**Keywords:** cell signaling, endothelial cell, macropinocytosis, PAI1, PAI1/uPA, plasminogen activator inhibitor 1, serpine1

## Abstract

Plasminogen activator inhibitor 1 (PAI1) is a secreted protease inhibitor, but accumulating evidence supports a role for PAI1 in regulating intracellular processes. PAI1 is secreted in an active conformation that serves as a bait for plasminogen activators (PA). Interaction between PAs and PAI1 results in a covalent linkage. PAI1/PA bind LRP1 and uPAR on the cell surface, triggering internalization and degradation. However, it was recently shown that exogenous PAI1 directly interacts with endothelial nitric oxide synthase inside endothelial cells. Our objective was to characterize the non-degradative pathway for PAI1 internalization. We use a hemagglutinin (HA)-tagged construct of PAI1 to track endocytosis of exogenous PAI1 in cultured endothelial cells via Western blotting of cell lysates and immunofluorescence microscopy. We demonstrate that PAI1-HA is actively internalized through a non-receptor-mediated mechanism, independent of clathrin-mediated endocytosis or caveolae. PAI1-HA internalization was partially inhibited by a derivative of amiloride, an inhibitor of macropinocytosis, though it was independent of Rac1 and CDC42, GTPases used for macropinocytosis. After internalization, PAI1-HA evades degradation for at least 3 h. By contrast, PAI1/uPA is degraded within 30 min of internalization. PAI1-HA internalization is independent of LRP1 and uPAR. We propose a model whereby PAI1/PA is endocytosed by LRP1- and uPAR-dependent endocytosis, whereas latent PAI can be internalized through a pinocytic-like mechanism and evade degradation, facilitating paracrine signaling.

## 1 | Introduction

Plasminogen activator inhibitor 1 (PAI1) is a secreted serine protease inhibitor that is elevated in numerous disease states including preeclampsia [1, 2], type 2 diabetes [3], and cardiovascular disease [4, 5], but its contributions to these diseases are not fully understood. PAI1 inhibits tissue- and urokinase-type plasminogen activators (tPA and uPA, respectively), thereby stabilizing fibrin clots [6] and promoting vascular fibrosis [7]. A canonical signal peptide at the N-terminus of PAI1 drives its secretion to the extracellular space, which is up-regulated by TNF*α*, TGF*β*, Angiotensin II, and hypoxia [8–11].

PAI1 is a suicide inhibitor of the plasminogen activators. It is secreted in an active conformation, which has an exposed reactive center loop that serves as a “bait sequence” for tPA/uPA. When tPA or uPA cleaves the bait sequence, PAI1 undergoes a conformational change in which the reactive center loop folds inward, trapping tPA/uPA, and a covalent interaction between PAI1 and the PA forms [6]. The active conformation is thermodynamically unstable. In vitro, the active conformation has a short half-life and converts to a latent conformation in which the reactive center loop is not accessible. Prior studies on the fate of extracellular PAI1 have focused on internalization of PAI1 when in complex with tPA/uPA. By monitoring ^125^I-labeled uPA complexed with PAI1, it was found that the complex binds cell surface receptors lipoprotein related peptide 1 (LRP1) and urokinase-type plasminogen activator receptor (uPAR), which triggers internalization and degradation of the radio-labeled substrate while LRP1 and uPAR are recycled to the cell surface [12–14]. Blocking LRP1 with receptor-associated protein (RAP) blocks PAI1/uPA internalization [13, 15].

In addition to its canonical role in the extracellular space, accumulating evidence suggests that PAI1 also alters intracellular signaling. PAI1 inhibits PI3K/Akt growth signaling, at least partially dependent on its interaction with LRP1 on the cell surface [16, 17], and interaction between PAI1 and LRP1 on the cell surface may provoke Janus kinase/signal transducer and activator of transcription (JAK/STAT) activation, promoting endothelial migration [18]. In addition, direct intracellular activity by PAI1 has been demonstrated by several groups. Within the Golgi, PAI1 inhibits furin proprotein convertase, leading to a reduction in maturation of insulin receptor and ADAM17 [19]. Our group recently showed that PAI1 directly interacts with and inhibits endothelial nitric oxide synthase (eNOS) [20]. Moreover, using a C-terminal hemagglutinin tagged version of PAI1, it was found that exogenous PAI1 is internalized, exhibits localization signals in the Golgi and cytosol, and colocalizes with eNOS. Most recently, PAI1 was observed within the nucleus and found to bind DNA via chromatin immunoprecipitation, suggesting that PAI1 may directly regulate transcription [21]. Together, these findings demonstrate intracellular effects of PAI1, a secreted protein, and suggest that PAI1 can escape degradation upon endocytosis. However, the mechanism by which PAI1 is internalized but evades degradation is not known.

Thus, our objective was to characterize PAI1 internalization by endothelial cells. Here, we demonstrate that exogenous PAI1 is taken up by cultured endothelial cells through a macropinocytosis-like mechanism, which is distinct from PAI1/uPA and independent of uPAR and LRP1.

## 2 | Materials and Methods

### 2.1 | Cell Culture

Ea.hy926 cell line was purchased from the American Type Culture Collection (CRL-2922) and grown in Dulbecco’s Modified Eagle Medium (DMEM) (Gibco) containing 10% fetal bovine serum (FBS), penicillin (100 U/mL), streptomycin (0.1 mg/mL), glutamine (2 mM), and hypoxanthine-aminopterin-thymidine (HAT) as previously described [20]. Primary human umbilical vein endothelial cells (HUVECs) were obtained from the Yale University Vascular Biology and Therapeutics Core facility, plated on 0.1% gelatin-coated dishes in Endothelial Cell Growth Medium (Lonza), and used at passage 4. Human umbilical arterial endothelial cells (HUAECs) were obtained from PromoCell, grown in endothelial growth media, and used at passage 4. Human microvascular endothelial cells (MVECs) were a gift of Dr. Seth Guller, cultured in endothelial growth media, and used at passage 6. COS7 cells were cultured in DMEM supplemented with 10% FBS, penicillin (100 U/mL), streptomycin (0.1 mg/mL), and glutamine (2 mM).

### 2.2 | Transfection

For experiments with silencing RNA (siRNA), endothelial cells were grown to 70% confluence and transfected with 20 nM siRNA targeting PAI1 (Dharmacon, D-019376–01), LRP1 (Dharmacon, D-004721–01), uPAR (Dharmacon, D-006388–18), clathrin heavy chain (ThermoFisher), caveolin 1 (Invitrogen, 4 427 038), dynamin 2 (Invitrogen, 4 427 038), CDC42 (Dharmacon, J-005057–05), Rac1 (Dharmacon, J-003560–14), or control non-targeting pool siRNA (Dharmacon, D-001206–14) using Lipofectamine RNAiMax (ThermoFisher Scientific) in Opti-MEM medium (ThermoFisher Scientific). After 6–8 h, the media was replaced with complete media containing 10% FBS as above. Experiments were conducted after 48 h of RNA silencing.

A construct for over-expression of PAI1 with a C-terminal hemagglutinin (HA) tag was previously generated [20]. COS7 cells were transfected with 1 μg plasmid DNA (pcDNA3 empty vector or PAI1-HA) per 10 mm culture dish using Lipofectamine 2000 (ThermoFisher Scientific) in Opti-MEM medium as previously described. After 8 h, the media were replaced with complete media containing 10% FBS as described above. After 48 h of incubation, PAI1-HA or pcDNA3 enriched spent media were collected and strained through a 40 μm cell-strainer. Integrity and composition of the samples were confirmed by Western blotting against the HA tag and PAI1. Aliquots were used fresh or frozen at −80°C for later use.

### 2.3 | PAI1-HA Endocytosis

Endothelial cells were grown to 90% confluence in 6 well dishes. PAI1-HA enriched media was added to each well for 2 h unless otherwise stated and cells were incubated at 37°C. In some experiments, cells were pre-treated with Dynasore (80 μM, Sigma), 5-(N-Ethyl-N-isopropyl) amiloride (100 μM, Sigma), nocodazole (10 μM, Sigma), phorbol myristate acetate (PMA) (100 nM), or MG132 (10 μM, Selleck) for 30 min prior to addition of enriched media. Human uPA/PAI1 was purchased from Innovative Research and added to cells at 3.3 μg/mL. In some experiments, recombinant receptor associated protein (200 nM, Enzo) was added for 15 min prior to addition of recombinant PAI1 or uPA/PAI1. Cells were then washed with ice-cold PBS and lysates collected in Laemlli buffer. For acid wash, cells were washed with ice cold glycine wash buffer pH 3 twice followed by PBS whereas controls were washed with ice cold PBS three times.

### 2.4 | Immunoblotting

Samples were boiled in loading buffer and separated by sodium dodecyl sulfate polyacrylamide gel electrophoresis (SDS-PAGE) and then transferred to 0.45 μm nitrocellulose membranes (Bio-Rad). The following primary antibodies were used: HSP90 (Santa Cruz, sc-13 119), HA (Roche clone 3F10), LRP1 (Cell Signaling, 64099S), uPAR (Abcam, ab103791), Clathrin heavy chain (BD), caveolin 1 (BD Biosciences), Dynamin 2 (Abcam, ab3457), CDC42 (Abcam, ab187643), Rac1 (EMD Millipore, 05–389), uPA (Invitrogen PA5–34638) at 1:1000 dilution. The appropriate Li-COR secondary antibodies were used at 1:10000. Western blotting quantification was performed in ImageJ. Briefly, mean gray value was measured for each band and the surrounding background. Pixel density was inverted and the background subtracted from the band, which was then normalized to the loading control (HSP90). For assessment of inhibitors and siRNA, HA signal at 2 h was used as the reference and the percent PAI1-HA uptake was calculated as the percent difference between signal in the treatment group compared to the control, divided by the control.

### 2.5 | Immunofluorescence Microscopy

HUVECs grown on coverslips coated with 0.1% gelatin were treated with media enriched for PAI1-HA for 2 h or as otherwise stated. Cells were washed with ice cold PBS, fixed with 4% formaldehyde for 10 min at room temperature, permeabilized with 0.1% Triton X100 for 10 min at 4°C, and incubated in blocking buffer (PBS containing 3% bovine serum albumin) for 1 h at room temperature. Slides were incubated with primary antibodies at a dilution of 1:100 overnight at 4°C unless otherwise stated. The following primary antibodies were used: EEA1 (Abcam, AB109110, 1:500), LAMP1 (Abcam, RM1217), LC3–647 (Proteintech, CL647–14600, 1:50). In some experiments, lucifer yellow (Cayman Chemicals) (1 mg/mL) or Transferrin-FITC (Invitrogen) (50 μg/mL) were added to cells in pcDNA3-enriched media or PAI1-HA-enriched media. Slides were washed and incubated with the appropriate AlexaFluor-conjugated (Life Technologies) secondary antibody diluted 1:1000 for 1 h. Hoechst or DAPI nuclear antibody stain was applied, and coverslips were mounted on slides with mounting media. Samples were visualized using Keyance BZ-X series and LeicaSP5 confocal microscopes. To determine PAI1-HA internalization, 10 random fields of view containing endothelial cells with normal morphology were captured with the same exposure time and gain settings. Each image was analyzed in ImageJ. For each field of view, regions of interest (ROI) were drawn around each cell in a brightfield image, then the integrated density was measured for each ROI in the appropriate fluorescent channel. To count cells positive for PAI1-HA uptake, the threshold function was used to convert the PAI1-HA channel to binary, and the percentage of positive cells was measured. To assess colocalization with endocytic markers, the JACoP plug-in was used [22].

### 2.6 | Statistical Analysis

Statistical analysis was performed in GraphPad Prism 9. Results are presented as scatterplots with horizontal lines at the median. Differences between two groups were compared by unpaired parametric or non-parametric *t*-tests as applicable. Experiments in which more than one comparison was tested were analyzed by one-way analysis of variance (ANOVA) followed by Dunnett’s multiple comparisons test or two-way ANOVA followed by Šídák’s multiple comparisons test. *p* < 0.05 was considered significant.

## 3 | Results

### 3.1 | Exogenous PAI1 Is Endocytosed by Endothelial Cells

**FIGURE 1 |:**
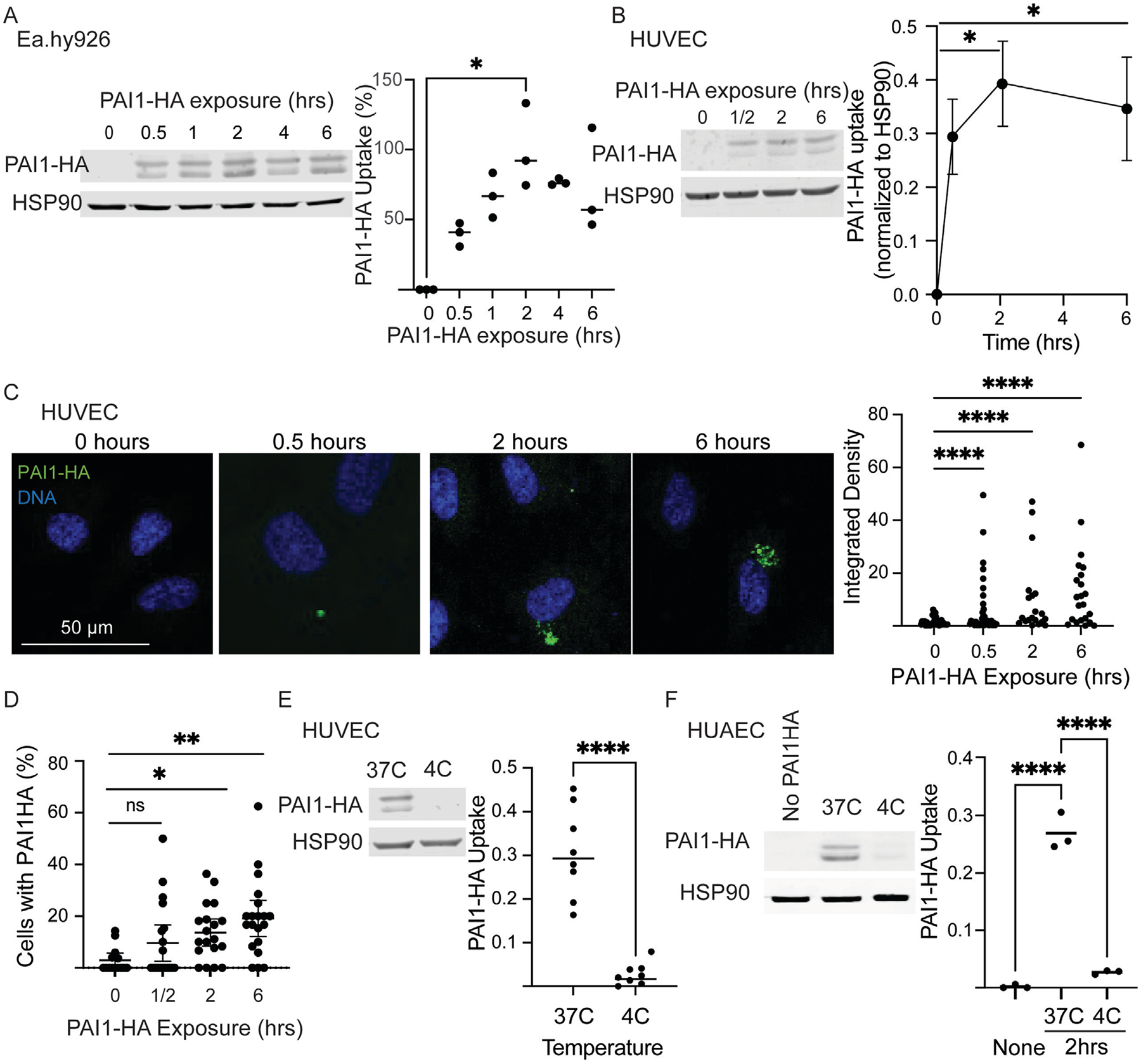
Plasminogen activator inhibitor-1 (PAI1) is actively internalized by endothelial cells. A. PAI1-HA enriched media was added to Ea.hy926 cells (A) and HUVECs (B) and incubated for the indicated length of time. Cells were washed, and cell lysates measured by Western blotting, probing for the HA tag with primary antibody. The amount of PAI1HA signal was quantified in relation to HSP90 loading control. Graph represents mean (SEM) from 3 independent experiments. C. PAI1-HA enriched media was added to HUVECs for 0, 30 min, 2, or 6 h. Cells were fixed and PAI1-HA visualized using primary antibody against the HA tag. Fluorescent intensity was measured as the integrated density of PAI1-HA channel. Differences among groups were assessed using one-way ANOVA with post hoc comparison of each time point to 0 h. D. In the experiments described in (C), it was noted that not all cells contained puncta of internalized PAI1-HA. In each field of view, the percentage of cells containing PAI1-HA were counted. One-way ANOVA with post hoc comparison to 0 h was performed. E. PAI1-HA was added to HUVECs and incubated at 37°C or 4°C for 2 h, followed by lysis and assessment of internalized PAI-HA via Western blotting. F. PAI1-HA was added to human umbilical arterial endothelial cells (HUAECs) for 2 h at 37°C or 4°C for 2 h, followed by lysis and assessment of internalized PAI-HA via Western blotting. Difference was assessed via *t*-test, ns, not significant. Differences were compared using one-way ANOVA with post hoc analysis of the shown comparisons. NS, not significant. *denotes *p* < 0.05. ** denotes *p* < 0.01. **** denotes *p* < 0.0001.

PAI1 with a C-terminal hemagglutinin tag was over-expressed in COS7 cells to generate PAI1-HA enriched, spent media. PAI1-HA media was added to Ea.hy926 endothelial cells (Figure 1A) or human umbilical vein endothelial cells (HUVECs) (Figure 1B) in culture. Exogenous PAI1-HA is taken up by endothelial cells within 30 min and reaches a steady state in the cell by 2 h. To confirm PAI1-HA was internalized, cells were exposed to PAI1-HA, fixed, and visualized by immunofluorescence microscopy to the HA tag to identify exogenous PAI1 (Figure 1C). However, only a subset of cells demonstrated internalized PAI1-HA, reaching a mean of 11% ± 10% of cells at 2 h of incubation (Figure 1D). To improve sensitivity for PAI1-HA, tyramide amplification was used. Using this approach, PAI1-HA was detectable in 46% ± 8% of cells (not shown). To test whether this was an active process, PAI1-HA was added to endothelial cells at 4°C for 2 h, which demonstrated minimal PAI1-HA internalization compared to incubation at 37°C (Figure 1E). To assess whether PAI1-HA internalization is specific to venous endothelial cells, PAI1-HA internalization was also assessed in human umbilical artery endothelial cells (HUAECs). HUAECs also internalize PAI1-HA at 37°C but not at 4°C. These findings confirm that PAI1-HA is actively internalized by endothelial cells.

### 3.2 | PAI1-HA Uptake Is Independent of Classical Endocytic Mechanisms

**FIGURE 2 |:**
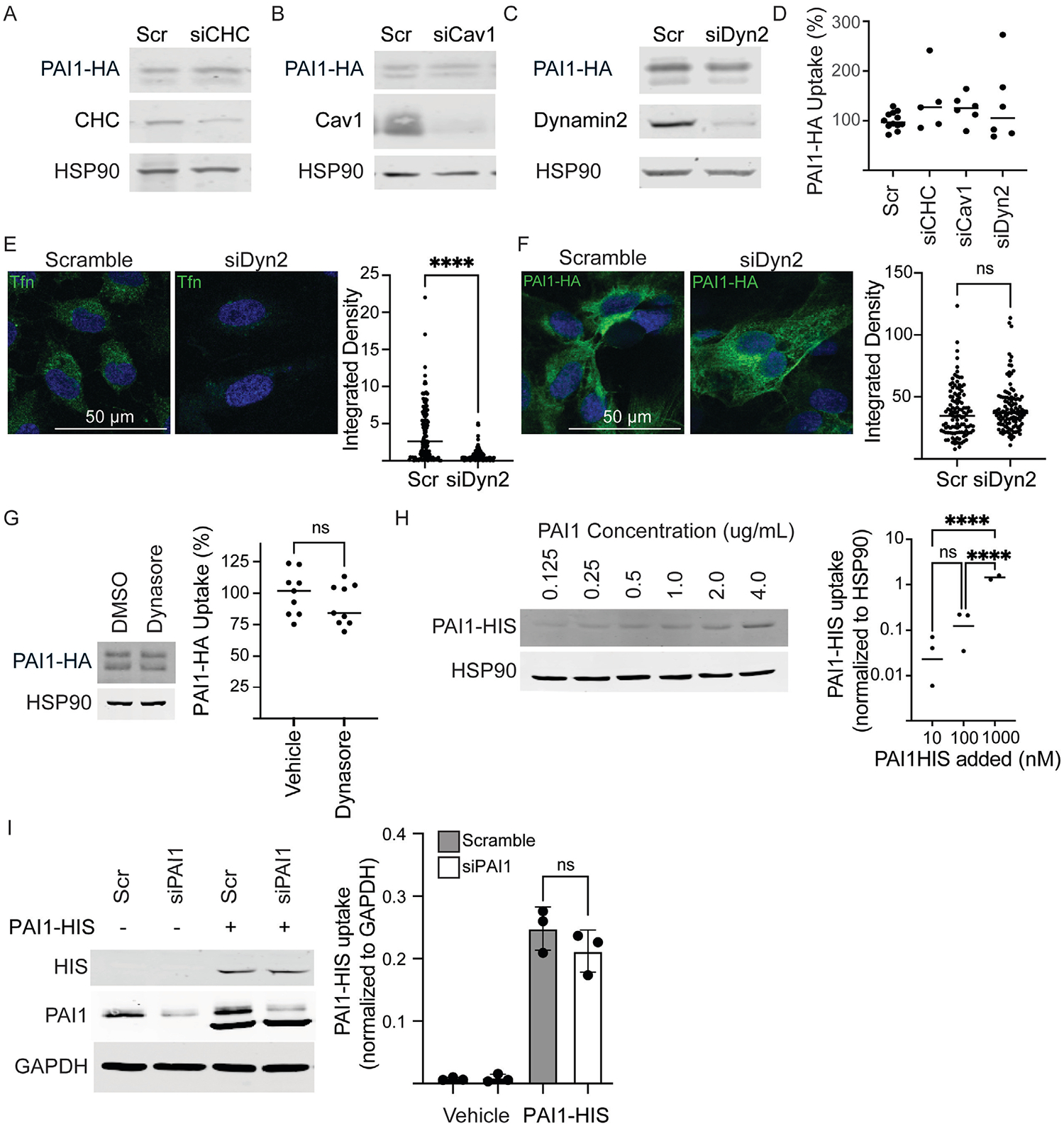
PAI1-HA internalization is independent of clathrin-mediated endocytosis and caveolae. HUVECs were transfected with siRNA to silence clathrin heavy chain (CHC) (A), caveolin 1 (Cav1) (B), or dynamin 2 (Dyn2) (C) for 48 h prior to addition of PAI1-HA enriched media for 2 h. Cells were lysed and internalized PAI1-HA was visualized by Western blotting using an anti-HA primary antibody. D. PAI1-HA internalization when endocytic machinery was silenced was compared to control treated siRNA cells and expressed as a percentage. Graph demonstrates individual experiments from at least three biological replicate experiments. One-way ANOVA was performed. All differences, *p* > 0.05. HUVECs were transfected with siRNA to silence Dynamin 2 for 48 h followed by addition of FITC-conjugated transferrin (E) or PAI1-HA (F) and fixed and prepared for IF using tyramide amplification. Silencing dynamin 2 significantly reduced transferrin internalization (E) but had no effect on PAI1-HA internalization (F). G. Pre-treatment of HUVECs with Dynasore, an inhibitor of Dynamin 2, for 30 min prior to addition of PAI1-HA enriched media had no effect on internalized PAI1-HA. Non-parametric Mann–Whitney test was used to assess difference. H. Recombinant purified PAI1-HIS was added to HUVECs at the specified concentrations for 30 min and internalization measured via Western blotting, normalized to HSP90 loading control. PAI1HIS internalization was not saturable up to 1 μM PAI1. I. HUVECs were treated with scramble control (Scr) or PAI1-specific siRNA. Recombinant PAI1-HIS or vehicle was then added for 2 h, 30 min, or 6 h. Internalized PAI1-HIS was visualized by Western blotting. One way ANOVA was used with post hoc comparisons to 100 nM. ns, not significant. **** denotes *p* < 0.0001.

To characterize the pathway by which PAI1-HA is internalized but evades degradation, we investigated the role of clathrin-mediated endocytosis and caveolae, two well-described endocytic pathways employed by endothelial cells, both of which use the GTPase Dynamin 2 to pinch off endocytic vesicles from the plasma membrane [23]. To investigate whether clathrin-mediated endocytosis or caveolae are used for PAI1-HA endocytosis, siRNA was used to silence clathrin heavy chain, caveolin-1, or dynamin2 prior to PAI1-HA addition. Despite efficient silencing of clathrin heavy chain, caveolin-1, and dynamin2, there was no decrease in PAI1-HA internalization as measured by semi-quantitative Western blotting (Figure 2A–D). Clathrin-mediated endocytosis is used for internalization of transferrin via the transferrin receptor [23]. Silencing Dynamin 2 significantly reduced transferrin internalization as visualized by IF microscopy (Figure 2E) but not PAI1-HA (Figure 2F). Similarly, pre-treatment of HUVECs with Dynasore, an inhibitor of Dynamin 2, did not alter PAI1-HA uptake (Figure 2G).

Receptor-mediated endocytosis is saturable based on the abundance of the receptor. To assess whether PAI1 internalization is saturable, recombinant PAI1 with a C-terminal HIS tag was added to HUVECs at increasing concentrations for 30 min, and internalization was assessed via Western blotting. Upon addition of PAI1-HIS from 1 nM to 1 μM, PAI1-HIS internalization was dose-dependent and not saturable (Figure 2H). To investigate whether PAI1 internalization is regulated by the abundance of PAI1 within the cell, PAI1 was silenced in HUVECs using siRNA. Cells were then exposed to recombinant PAI1 with a C-terminal histidine tag. There was no difference in exogenous PAI1-HIS internalization based on endogenous PAI1 expression (Figure 2I). Taken together, these results suggest that PAI1-HA internalization does not employ receptor-mediated endocytosis.

### 3.3 | PAI1-HA Uptake Has Features of Macropinocytosis

**FIGURE 3 |:**
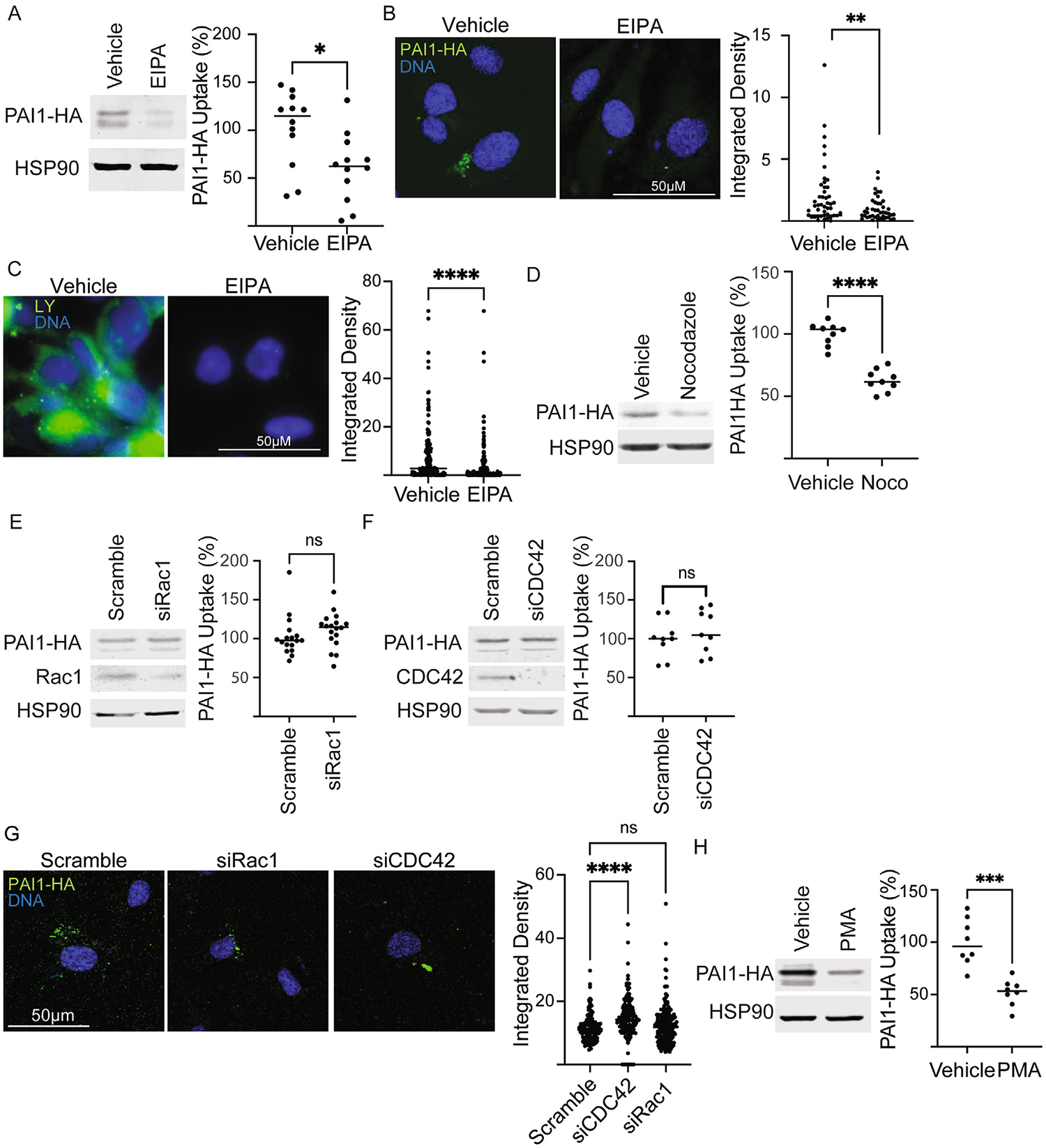
PAI1-HA internalization has features of macropinocytosis. A. HUVECs were pre-treated with EIPA for 30 min prior to addition of PAI1-HA enriched media for 2 h. Internalized PAI1-HA was visualized by Western blotting. Graph depicts results from four biological replicates. B. Cells were pre-treated with EIPA for 30 min prior to addition of PAI1-HA for 2 h. Internalized PAI1-HA was visualized by immunofluorescence to HA tag. C. Cells were pre-treated with EIPA for 30 min prior to addition of lucifer yellow (LY) for 30 min. D. Cells were pre-treated with nocodazole (Noco) for 30 min prior to addition of PAI1-HA enriched media, and internalization was assessed via Western blotting. Cells were treated with control siRNA or siRNA to silence Rac1 (E, G) or CDC42 (F, G) for 48 h, followed by addition of PAI1-HA enriched media. Internalized PAI1-HA was assessed via Western blotting (E, F) or immunofluorescence to HA tag (G). H. Cells were treated with phorbol myristate acetate (PMA) (100 nM) for 30 min prior to addition of PAI1-HA enriched media. Internalized PAI1-HA was assessed via Western blotting. Differences were assessed by *t*-test. NS, not significant. * denotes *p* < 0.05. ** denotes *p* < 0.01. **** denotes *p* < 0.0001.

Non-saturable endocytic kinetics suggests a receptor-independent uptake mechanism. Endothelial cells use macropinocytosis for signaling of fibroblast growth factor (FGF) and vascular endothelial growth factor (VEGF) [24–26]. Amiloride is a Na^+^/H^+^ exchange inhibitor that inhibits macropinocytosis [27]. We hypothesized that PAI1-HA internalization could occur via macropinocytosis. To investigate whether amiloride inhibits PAI1-HA uptake, endothelial cells were pre-treated with EIPA, a derivative of amiloride, for 30 min prior to addition of PAI1-HA enriched media for 2 h. Western blotting of cell lysates demonstrated that pre-treatment with EIPA led to approximately 50% less PAI1-HA internalization compared to vehicle-treated cells (Figure 3A). EIPA blocked PAI1-HA uptake as seen by IF (Figure 3B). EIPA also blocked internalization of lucifer yellow, a marker of fluid phase pinocytosis. Pretreatment with nocodazole, an inhibitor of microtubule formation and macropinocytosis [28] also reduced PAI1-HA internalization approximately 50% (Figure 3D). Macropinosomes are closed off into pinocytic vesicles through activity of small GTPases including Rac1 and CDC42 [27, 29–33]. However, silencing of neither Rac1 nor CDC42 reduced PAI1-HA internalization (Figure 3E–3G). Lastly. PMA is reported to stimulate macropinocytosis [28]. When HUVECs were pre-treated with PMA, we found reduced PAI1-HA uptake (Figure 3H). These findings suggest that PAI1-HA uptake has features similar to but distinct from macropinocytosis.

### 3.4 | PAI1-HA Can Evade Rapid Degradation in Endothelial Cells

**FIGURE 4 |:**
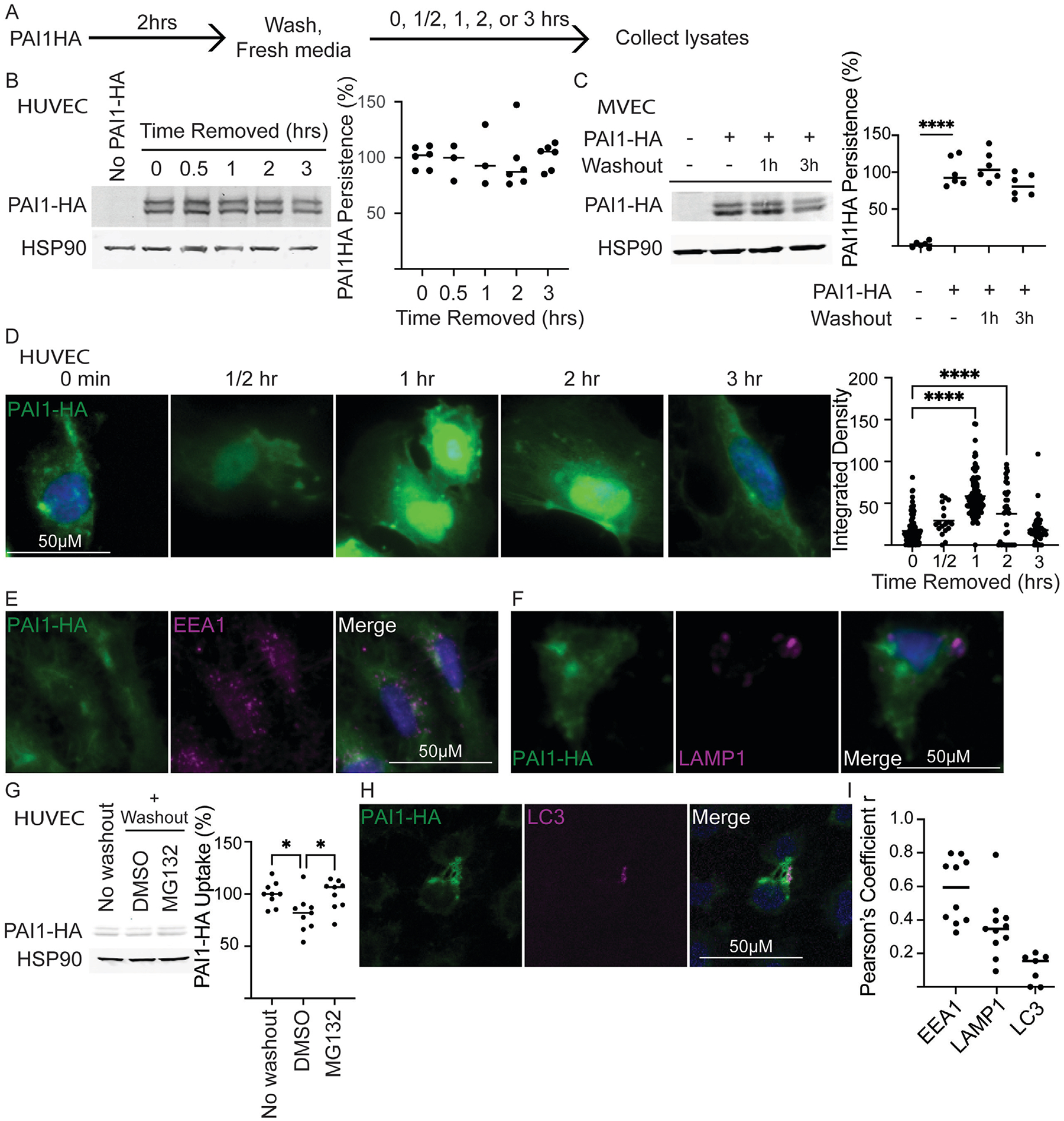
PAI1-HA evades degradation after internalization. A. Schematic depicting experimental design for panels B-D. PAI1-HA enriched media was added to cells for 2 h. Cells were then washed and given fresh complete growth media. Cell lysates were collected at different time points. Internalized PAI1-HA was assessed in HUVECs (B) and human microvascular endothelial cells (C) via Western blotting. Persistence was expressed as a percentage in relation to the intensity at the 0 time point. D. Internalized PAI1-HA in HUVECs was visualized by immunofluorescence microscopy to the HA tag using tyramide amplification. Differences among groups were determined by one-way ANOVA with post hoc test for multiple comparisons. E-G. Cells treated with PAI1-HA enriched media for 30 min were fixed and stained for HA tag and EEA1 (E) or LAMP1 (F). Tyramide amplification was used to enhance the HA signal. Cells were visualized by immunofluorescence microscopy. Colocalization shown in white. G. HUVECs were exposed to PAI1HA for 2 h followed by 3 h washout period with MG132 or DMSO control. Internalized PAI1-HA was assessed via Western blotting. H. Cells treated with PAI1-HA enriched media, fixed, and stained for HA tag and LC3. I. Colocalization was assessed via Pearson correlation coefficient. NS, not significant. * denotes *p* < 0.05. **** denotes *p* < 0.0001.

For exogenous PAI1-HA to exert intracellular effects, we hypothesize that it can evade degradation after internalization. To investigate this question, PAI1-HA enriched media was added to HUVECs for 2 h; the media was then removed, cells washed, and fresh complete growth media was added (Figure 4A). Cell lysates were collected at different times after removal of PAI1-HA. PAI1-HA persisted in endothelial cells for at least 3 h (Figure 4B). To investigate the cell type specificity of this phenomenon, we repeated the experiment in human glomerular microvascular endothelial cells (MVECs) (Figure 4C), which confirmed PAI1-HA is taken up and evades degradation. Persistence of internalized PAI1-HA in HUVECs was also visualized by immunofluorescence microscopy (Figure 4D). These data suggest that exogenous PAI1-HA can be internalized and evade degradation over several hours.

To better characterize the endocytic characteristics of PAI1-HA internalization, HUVECs were exposed to PAI1-HA and slides were prepared for colocalization between PAI1-HA and endocytic markers. PAI1-HA exhibited moderate colocalization with EEA1, a marker of early endosomes (Pearson coefficient 0.57 ± 0.20) (Figure 4E,I). In contrast, PAI1-HA did not colocalize with LAMP1, a marker of lysosomes (Pearson coefficient 0.35 ± 0.18) (Figure 4F,I). To investigate whether autophagy contributes to PAI1-HA turnover, HUVECs were treated with PAI1-HA enriched media for 2 h, followed by removal and washout period of 3 h. There is a reduction in PAI1-HA in cell lysates after the washout period that is blocked by MG132, a proteasomal inhibitor (Figure 4G), suggesting that ultimately PAI1-HA is degraded by proteasomal degradation. To investigate whether PAI1-HA is trapped in autophagosomes, colocalization of internalized PAI1-HA was visualized with LC3, a proteasome marker. There was no colocalization of LC3 with PAI1-HA (Pearson coefficient 0.08 ± 0.11) (Figure 4H,I). These data support the hypothesis that PAI1-HA evades lysosomal degradation upon internalization but is ultimately degraded by the proteasome.

### 3.5 | PAI1-HA Internalization Is Distinct From PAI1-uPA Internalization

**FIGURE 5 |:**
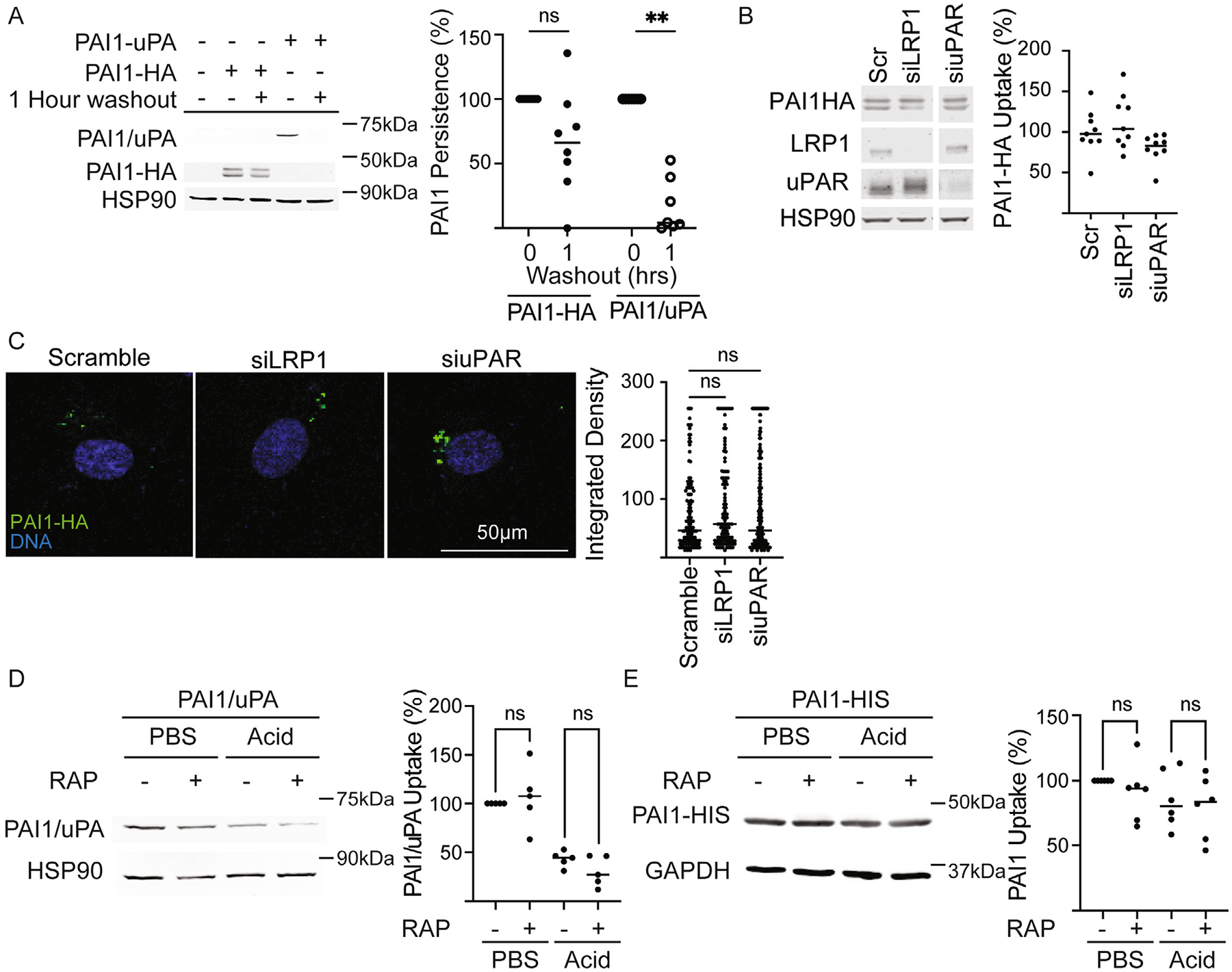
PAI1-HA is internalized via a different mechanism than PAI1/uPA. A. PAI1-HA enriched media or purified human PAI1/uPA complex were added to endothelial cells for 30 min. Cells were either washed and lysed at that time or washed and incubated with complete growth media for 1 h (washout) prior to collecting lysates. Internalized/persistent PAI1 was determined by Western blotting to the HA tag or to PAI1 at the predicted molecular weight of PAI1/uPA normalized to GAPDH. Four biological replicates were performed in duplicate or triplicate. Differences were assessed using 2-way ANOVA with Sidak’s multiple comparison’s test. Results graphed as percentage of PAI1 compared to the initial timepoint. B. HUVECs were transfected with siRNA to silence LRP1 or uPAR for 48 h prior to addition of PAI1-HA enriched media for 2 h. Cells were lysed and internalized PAI1-HA was visualized by Western blotting using an anti-HA primary antibody. Differences were compared using one-way ANOVA. C. As in B, but cells were fixed and stained with primary antibody to HA tag and visualized by IF. D. HUVECs were pre-treated with receptor associated protein (RAP) or vehicle for 30 min prior to addition of recombinant PAI1/uPA. After 1 h incubation, cells were washed with PBS or glycine buffer pH 3 and lysates were run on Western blot. E. As in D, but cells were treated with recombinant PAI1-HIS. Differences were compared using one-way ANOVA. NS, not significant. * denotes *p* < 0.05. ** denotes *p* < 0.01.

While Figure 4 demonstrates that PAI1-HA persists in endothelial cells, previous work has demonstrated that PAI1/uPA is degraded rapidly upon internalization [34]. To test whether latent PAI1 and PAI1/uPA experience the same fate, PAI1-HA and PAI1/uPA complex (covalent complex) were added to endothelial cells for 30 min. After incubation, cells were either collected immediately or washed and replaced with fresh growth media for 1 h before lysis. PAI1-HA is internalized within 30 min and persists in cells after a 1 h washout period. PAI1/uPA is also detectable in cell lysates after a 30 min incubation period but is no longer detectable in lysates after a 1 h washout period (Figure 5A). This suggests that internalized latent PAI1 has a distinct fate from PAI1/uPA.

PAI1/uPA internalization is dependent on cell surface receptors LRP1 and uPAR [13, 34, 35]. To test whether PAI1 endocytosis and persistence are dependent on LRP1 and uPAR, endothelial cells were transfected with siRNA that was non-targeting (Scramble) or co-transfected with siRNA to silence LRP1 and uPAR. Efficient silencing of LRP1 and uPAR was confirmed by Western blot 48 h after transfection but did not alter PAI1-HA internalization as visualized by Western blotting (Figure 5B) or immunofluorescence microscopy (Figure 5C).

Prior work demonstrating the role of LRP1 in PAI1/uPA endocytosis used the 39 kDa receptor associated protein (RAP), which sterically blocks LRP1 [13, 15]. To further confirm whether latent PAI1 internalization differed from PAI1/uPA internalization, cells were pre-treated with recombinant RAP prior to addition of PAI1. There was minimal reduction in PAI1/uPA internalization with RAP pre-treatment (Figure 5D). However, to test whether detectable PAI1/uPA remained associated with the cell surface despite internalization being blocked, cells were washed with acidic glycine buffer prior to lysis to remove surface-associated proteins. There was a significant reduction in PAI1/uPA in cell lysates after acid wash, indicating that much of the PAI1/uPA was not actually internalized to the cell. Addition of RAP led to a small additional reduction in PAI1/uPA cell lysates. In contrast, there was no difference in PAI1-HIS abundance in cell lysates after acid wash, and RAP had no effect on PAI1-HIS internalization (Figure 5E). These findings further demonstrate that latent exogenous PAI1 is internalized by endothelial cells to a greater extent than PAI1/uPA and minimally uses LRP1.

## 4 | Discussion

PAI1, which is primarily characterized for its role in negatively regulating fibrinolysis, is increasingly understood as a bio-marker of metabolic syndrome and acute inflammatory states such as acute respiratory distress syndrome [36]. In addition to inhibiting tPA and uPA, it also inhibits other serine proteases [37]. Biological activity of intracellular PAI1 has recently been demonstrated by several independent groups [19–21]. It was not known from these studies whether the PAI1 exerting intracellular effects was via endogenous PAI1 on its way through the protein secretion pathway, via interaction with a transmembrane receptor such as LRP1, or through internalization of exogenous PAI1. Garcia et al. generated a PAI1 construct with C-terminal HA tag and demonstrated that PAI1-HA colocalizes with eNOS in endothelial cells. Moreover, PAI1 has recently been demonstrated to have nuclear localization and to act as a transcription repressor in bladder cancer [21]. However, it is unknown how PAI1, which has a signal peptide and is secreted into the extracellular space, is endocytosed and evades lysosomal degradation.

Here, we sought to explore how exogenous PAI1 is internalized by endothelial cells. We confirm that exogenous PAI1 added to endothelial cells in vitro is actively internalized and colocalizes with EEA1, a marker of early endosomes. Surprisingly, using both chemical inhibitors and genetic silencing, we found that PAI1 entry was independent of clathrin-mediated endocytosis or caveolae. PAI1 reached a steady-state in endothelial cells by 2 h of exposure, but internalization within 30 min was not saturable up to 1 μM of PAI1. Moreover, alterations in endogenous PAI1 expression did not affect internalization of exogenous PAI1, consistent with a non-receptor mediated process. PAI1 internalization was inhibited by EIPA and nocodazole, suggesting a macropinocytosis-like mechanism. However, unlike macropinocytosis, PAI1 internalization was not dependent on Rac1 or CDC42, which are described as key GTPases for driving macropinocytic ruffles, and was not stimulated by PMA. Importantly, this endocytic mechanism allows PAI1 to evade lysosomal degradation, persisting in endothelial cells for at least 3 h before proteosomal degradation. By contrast, PAI1/uPA complex is only partially internalized and is rapidly degraded upon internalization. Furthermore, PAI1/uPA internalization uses LRP1, whereas latent PAI1 internalization is not affected by blocking LRP1. Based on our findings, we propose a model whereby PAI1 can exert differential effects on endothelial cells depending on its conformation. PAI1 in its latent conformation (e.g., PAI1-HA, PAI1-HIS) is internalized through a macropinocytosis-like mechanism and traffic to eNOS [20], whereas PAI1/uPA interacts with LRP1 and is degraded upon internalization.

Our finding that PAI1-HA is internalized via macropinocytosis is in contrast with the conventional understanding that PAI1 endocytosis occurs through direct interaction with LRP1 and uPAR on the cell surface. PAI1 in its active conformation acts as a bait for its target serine protease; cleavage of its target sequence results in a conformational change that traps the serine protease [38]. Under physiologic conditions, the active conformation is unstable with a half-life of 1–2 h [6]. Prior studies that demonstrated the role for LRP1 and uPAR have tracked the fate of the PAI1-uPA/tPA complex [13, 39, 40]. However, the active conformation of PAI1 that can interact with tPA or uPA is thermodynamically unstable. PAI1 exists in its active conformation for a very short half-life and rapidly folds into its latent conformation, which is unable to bind uPA/tPA. Our recent study demonstrated that PAI1-inhibition of eNOS can occur with PAI1 in its latent or active conformation [20]. The conformational status was not directly studied by Bernot et al. but the acidic environment of the Golgi is anticipated to stabilize the active conformation of PAI1 [19]. The conformational state was not addressed in Furuya et al. [21]. Interestingly, during our time course experiments, PAI1 did appear to develop a nuclear localization, as described in Furuya et al. we propose that PAI1 bound to plasminogen activators forms the ternary complex with uPAR and LRP1 on the cell surface, is internalized, and routed for lysosomal degradation as previously described, whereas unbound, latent PAI1 is internalized by macropinocytosis and escapes rapid degradation. In this way, the endocytic mechanism by which PAI1 is internalized could have differential signaling effects, allowing the endothelial cell to respond to its extracellular environment.

Macropinocytosis is a non-specific internalization process mediated by membrane ruffles driven by actin polymerization and invagination of the extracellular fluid and its contents. Prior reports showed that endothelial cells use macropinocytosis to sense and respond to extracellular signals. Endothelial cells internalize fibroblast growth factor 2 (FGF2) via macropinocytosis [24]. Basagiannis and colleagues found that when vascular endothelial growth factor (VEGF) receptor is internalized via macropinocytosis with VEGF, angiogenesis and migration are promoted, whereas constitutive recycling of VEGFR occurs via clathrin-mediated endocytosis [26]. Moreover, microtubule activity is required for VEGF-induced endothelial cell migration [41]. Lastly, other membrane receptors such as FGFR1, transforming growth factor β, and epidermal growth factor receptor have been reported to be internalized by different mechanisms under different cellular conditions [25].

While we found PAI1 internalization to share characteristics of macropinocytosis, the exact mechanism is unclear. It is surprising that silencing Rac1 or CDC42 did not decrease PAI1-HA uptake as visualized by Western blotting. One possible explanation is that endothelial cells have promiscuity with regards to GTPase activity and can use other GTPases depending on availability within the cell. Alternatively, because genetic silencing did not eliminate Rac1 protein expression, our findings may reveal that small amounts of GTPase are sufficient to support actin polymerization.

Our data suggest that PAI1 internalization is not receptor-mediated based on non-saturable kinetics and clathrin- and dynamin 2-independence. However, it is possible that at higher concentrations of PAI1, we could detect saturation. Given that PAI1’s concentration in plasma is approximately 700 pM [1], at lower concentrations, there could be a receptor-mediated process that we are not detecting in these experiments. For example, PAI1 is predicted *in silico* to interact with TMPRSS7, a transmembrane serine protease [42].

Because of the in vitro nature of this work, there are some limitations to our findings. To track the fate of exogenous PAI1, we used constructs with a C-terminal hemagglutinin tag or HIS tag, which could be probed with primary antibody. While these tags are small (HA 1.1 kDa, HIS 840 Da), a limitation to this approach is that the tagged protein could traffic differently from native PAI1 without a tag. Another limitation of this work is that all experiments were conducted using a monolayer of endothelial cells without flow. How the kinetics of PAI1 internalization could work in 3D systems under shear stress is unknown and is an important future direction of this work.

PAI1 has been assumed to contribute to disease by stabilizing fibrin networks, thereby promoting fibrosis. Increasing data suggest that PAI1’s intracellular activities may also have pathologic effects. By inhibiting furin proprotein convertase in the Golgi apparatus, PAI1 reduces insulin receptor and ADAM17 production, a proposed mechanism for a mechanistic role of PAI1 in Type 2 diabetes mellitus [19]. PAI1 appears to contribute to hypertension and fibrosis from chronic L-NAME exposure, as PAI1 knockout mice are resistant to the phenotype [43, 44], and it is reduced with a small molecule inhibitor of PAI1 [7]. Future work will investigate the effect of exogenous PAI1 on endothelial dysfunction in these diseases.

We propose a model in which PAI1 internalization can follow different pathways depending on its conformational state, thereby exerting differential effects on endothelial cells. Future work will compare the physiologic impact of PAI1 in its active conformation, latent conformation, and in complex with PAI1/uPA. Based on the findings of Garcia et al. and this work, we suggest that latent PAI1 evades degradation and alters endothelial physiology. By contrast, if the system could be manipulated to shuttle PAI1 to the LRP1/uPAR degradative pathway, this could remediate some effects of elevated PAI1 in disease.

In summary, we demonstrate that PAI1 is actively internalized by endothelial cells in a receptor-independent fashion that allows it to evade degradation. Our recent finding that exogenous PAI1 inhibits eNOS activity suggests a direct intracellular mechanism for these findings [20]. Given the multiple mechanisms by which PAI1 contributes to pathology, it is critical to better understand its biology. The studies reported here suggest an alternate mechanism by which exogenous PAI1 can enter the intracellular space.

## Data Availability

The data that support the findings of this study are available in the Materials and Methods and Results of this article.
